# O030: Towards patient safety: health care professionals knowledge, attitude and practice of safe injection in public hospitals in Mekelle, Tigray, North Ethiopia

**DOI:** 10.1186/2047-2994-2-S1-O30

**Published:** 2013-06-20

**Authors:** HB Gebru

**Affiliations:** 1 Nursing, Mekelle University, Mekelle, Ethiopia

## Introduction

Safe injection in health facilities is a major health providers, patients and community problem. Health workers are at risk especially those in clinical practice.

## Objectives

The aim of this study was to assess knowledge, attitude and practice of health care providers on safe injection.

## Methods

This was an institution based study conducted from May –June, 2011 using simple random Sampling method. The data was collected by Nurses using a questionnaire, analyzed by SPSS version 16 and results are presented in tables and figures.

## Results

A total of 189 health care providers were interviewed and only 161 (85%) have knowledge about unsafe injection practice can lead to risks, 135 (71%) have accepted that to use leftover medications from single dose or single-use vial for another patient. In addition, 149 (79%) know that drug incorrectly administered at anatomical site can lead to infection. Also, 137 (72%) were used new and unopened needle and syringe for injection and reconstitution. Fifty (26%) and 80 (42%) of the health professionals didn’t use aseptic technique and didn’t receive training on injection safety respectively. Currently, 75 (40%) of the study subjects have been accidentally exposed to needle stick injury and 84 (44%) of the health professionals were exposed to needle stick injury in the past six months. Forty five (24%) still had the practice to recapping needles after an injection and in which 65 (34%) of them recap with one hand and 32 (17%)of them recap with two hands.

## Conclusion

It was confirmed that there is a gap of knowledge, attitude and practice of health care professionals towards safe injection practice and needle stick injury is highly prevalent in the study area. Hence, provision of short term refresher trainings to the health care professionals to prevent transmission of infection in a clinical setting is needed.

## Disclosure of interest

None declared.

